# Supplementary material for: The association between paternal characteristics and exclusive breastfeeding in Ghana
Source: PLoS One. 2021 Jun 3;16(6):e0252517. doi: 10.1371/journal.pone.0252517 (PMC8174696; doi:10.1371/journal.pone.0252517)
Supplement: Attachment — Submitted filename: Response to Reviewers.docx [file pone.0252517.s003.docx]

**Responses to academic editor and reviewers comments**

**Academic Editor**

When submitting your revision, we need you to address these additional requirements.

1. Please ensure that your manuscript meets PLOS ONE's style requirements, including those for file naming.

**Response:** We thank the academic editor for the comment and suggestion. We have revised the entire manuscript to ensure it meets PLOS ONE requirements.

2. We note that you have indicated that data from this study are available upon request. PLOS only allows data to be available upon request if there are legal or ethical restrictions on sharing data publicly.

**Response:** We apologise for the error. The Data Availability Statement should be modified to read “Data are available from the Measure Demographic and Health Survey Program (visit https://www.dhsprogram.com/data/ ) for researchers who meet the criteria for access to confidential data. The data is labeled Ghana: Standard DHS, 2014 and it can be accessed on the following link: <https://dhsprogram.com/data/dataset/Ghana_Standard-DHS_2014.cfm?flag=0>”.

3. In your revised cover letter, please address the following prompts:

a) If there are ethical or legal restrictions on sharing a de-identified data set, please explain them in detail (e.g., data contain potentially sensitive information, data are owned by a third-party organization, etc.) and who has imposed them (e.g., an ethics committee). Please also provide contact information for a data access committee, ethics committee, or other institutional body to which data requests may be sent.

**Response:** We have indicated in the revised cover letter that “The Measure Demographic and Health Survey program is authorised to distribute the 2014 Ghana Demographic and Health Survey data. If a researcher needs the data for research purpose, he/she is required to register with the Measure Demographic and Health Survey program to gain unrestricted access to the data”.

4. b) If there are no restrictions, please upload the minimal anonymized data set necessary to replicate your study findings as either Supporting Information files or to a stable, public repository and provide us with the relevant URLs, DOIs, or accession numbers. For a list of acceptable repositories, please see http://journals.plos.org/plosone/s/data-availability#loc-recommended-repositories.

**Response:** The datasets used for the analysis have been attached as Supplementary Information files and we have indicated in the revised cover letter that “We have also attached the files we analysed as Supplementary Information files” (last sentence on page 1).

5. For studies involving humans categorized by race/ethnicity, age, disease/disabilities, religion, sex/gender, sexual orientation, or other socially constructed groupings, authors should: 1) Explicitly describe their methods of categorizing human populations, 2) Define categories in as much detail as the study protocol allows, 3) Justify their choices of definitions and categories, 4) Explain whether (and if so, how) they controlled for confounding variables such as socioeconomic status, nutrition, environmental exposures, or similar factors in their analysis.

**Response:** We have described how we categorised characteristics of respondents which are social constructs and how we controlled for confounding variables in the revised cover letter as follows: “In our study, we used characteristics of respondents such as sex, age, ethnicity, and religion. We followed the categorization of these variables used in the 2014 Ghana Demographic and Health Survey manual. We checked for multicollinearity using Variation Inflation Factor (VIF) and highly correlated variables such as maternal place of residence and religion were excluded from the analysis” (page 2, paragraph 1).

**Reviewer 1**

1. Consider making the title more descriptive.

**Response:** We thank the reviewer for the suggestion. We have changed the wording of the topic to make it more descriptive. The revised topic is “The association between paternal characteristics and exclusive breastfeeding in Ghana” (page 1, line 1).

2. Rationale/justification for the study needs to be stronger. What specifically is this adding to the current literature

**Response:** We have modified the rationale/justification for the study in the revised manuscript. The study adds to the exclusive breastfeeding literature by identifying partner characteristics that significantly predict the practice of exclusive breastfeeding in Ghana by using a nationally representative sample. Please see page 4, lines 79-81, and page 5, lines 82-95.

3. If the analyses were adjusted, that needs to be explained and adjusted results should be listed as AORs instead of ORs

**Response:** The results were adjusted and they have been explained in the revised manuscript. The partner, mother, and infant characteristics were run together at the regression stage using the ENTER METHOD. All adjusted results have been changed from ORs to AORs. See page 2, lines 29-31, and page 11, lines 191-206.

4. Description of inclusion criteria should be more specific in the abstract methods and methods section.

**Response:** We thank the reviewer for the comment. All infants under 6 months old with maternal and paternal characteristics were included in the present study. This, therefore, includes infants who were either exclusively breastfed or not. We have made the inclusion criteria more specific in the abstract (page 2, lines 23-24) and methods section (page 6, lines104-106) in the revised manuscript.

5. Results should be presented with 2 decimals; remove "95%" from methods

**Response:** The results section of the revised manuscript has been modified and the results are presented in two decimals where applicable (see from page 8, lines 158 - page 14, line 210). In addition, we have removed the” 95%” from the methods section and replaced it with “a significance level of 0.05” (page 8, lines 153-154).

6. Conclusion in abstract should be more specific. How do these findings help? How can they inform policies and interventions? What should public health professionals do differently in Ghana based on your findings?

**Response:** We have revised the conclusion in the abstract and the main manuscript to include what public health professionals can do differently. Public health professionals can adopt a couple-oriented approach to educate and counsel both mothers and partners on the importance of exclusive breastfeeding in Ghana (page 2, lines 34-36, and page 19, lines 319-320).

7. Are all authors in the same institution? If yes, that should be clarified on the title page

**Response:** At the time of submission of the manuscript, all authors were from the same institution. Currently, the first and third authors have moved to a different institution (the University of Environment and Sustainable Development). This has been clarified on the title page (page 1, lines 7-10).

8. Line 18 should read sub-Saharan

**Response:** We have modified the background of the abstract in the revised manuscript and the phrase “sub Saharan” has been deleted from it (page 1, lines 18-19).

9. Line 43 Odd to reference EBF as an intervention; consider calling it an "essential practice"

**Response:** We agree with the comment of the reviewer. We have changed the word “intervention” in the introduction to “practice” in the revised manuscript (page 3, line 39). Also, we have changed “exclusive breastfeeding” to “EBF” almost throughout the entire revised manuscript.

10. Line 46 Define WHO and UNICEF at first use

**Response:** We have defined “WHO” and “UNICEF” in the revised manuscript (page 3, lines 42-43).

11. Line 56 Provide reference for this claim

**Response:** Thanks for the observation. We have provided Olufunlayo et al., 2019 as a reference to support our claim on page 3, line 51.

12. Line 57-58 Who is saying they should be? Be sure to state if this is the WHO goal

**Response:** In the revised manuscript, we have indicated who made the estimation and it now reads “The WHO estimates that at least 50% and 70% of infants aged under 6 months globally should be exclusively breastfed by 2025 and 2030 respectively” (page 3, line 52).

13. Line 62 Would be interesting if you could discuss why the increase in EBF between 2003-2008; were there certain interventions that were successful but possible excluded partners? This would be a good place to improve your justification and rationale for this study

**Response:** In the revised manuscript, we have included policies and programmes that may have contributed to the increase in Ghana’s EBF rate between 2003 and 2008 as follows: “The increase in Ghana’s EBF rate can be attributed to the implementation of policies and programmes such as the Breastfeeding Promotion Regulations 2000 [14], Imagine Ghana Free of Malnutrition [15], and Infant and Young Child Feeding Strategy [16]. These policies and programmes highlighted the importance of practicing exclusive breastfeeding. For instance, the Breastfeeding Promotion Regulations 2000 prohibits the sale and promotion of infant formula or any other product marketed as appropriate for feeding infants less than six months of age in health facilities or public places [14]. It is worth noting that these policies and programmes placed less emphasis on couple-oriented approaches to enhance exclusive breastfeeding” (page 5, line 55 to page 6, line 61).

14. Line 62-63 Also, discuss possibilities for why EBF decreased again in 2014

**Response:** We have explained plausible reasons for the decline in EBF in 2014 in the revised manuscript (page 4, lines 64-70).

15. Line 71 How are partners defined here? Since this is a key point of your study, should clarify who is considered a partner using the DHS data

**Response:** We defined partners as the biological father of the child irrespective of the marital status (page 4, lines 79-80).

16. Line 91-107 Too much discussion of the DHS methods; you can simply reference the DHS manual that explains all these details

**Response**: We agree with the comment of the reviewer. We have reduced the discussion of the DHS methods and have referenced the DHS manual for detailed information (page 5, lines 102-103).

17. Line 105-106 contradicts your findings. You state that 613 were identified as exclusively breastfed? So did you only include infants who were exclusively breastfed? This contradicts your coding of 0 and 1; please explain and clarify the inclusion and exclusion criteria

**Response**: Thank you for the observation. The 613 included all infants under six months who were either exclusively breastfed or not. However, with the inclusion of other variables suggested by the reviewers the sample size has currently reduced to 180. This includes all infants under six months with information on both maternal and paternal characteristics. It also includes those who were exclusively breastfed as well as those who were not exclusively breastfed. See from page 5, line 103 to page 6 line 106.

18. Line 119-129 Need rationale for the inclusion of these partner characteristics; it's concerning that a large independent variable is missing from this study; intimate partner violence and its' many forms should be considered to be included; this is largely a partner characteristic that is known to influence exclusive breastfeeding; also the number of children the partner has

**Response:** We acknowledged that some partner characteristics were missing in the first manuscript. This has been rectified and we have included other partners’ characteristics such as husband desire for children, religion, ethnicity, place of residence, partner’s attitude towards wife beating, and number of children by partner in the revised manuscript. See from page 6, line 118 to page 7, line 133.

19. Line 132-137 Were antenatal visits included? If yes, please add. If not, please justify why they were not included.

**Response**: Antenatal visits was included in the first manuscript we submitted for review and it is also included in the revised manuscript (page 7, line 142).

20. Line 138-155 This can be explained by adding it to one of your results tables; does not need to be listed out in the text.

**Response**: The section on the control variables has been summarised (page 7, lines 136-145).

21. Line 157 Data Analysis: Please clarify in detail how you addressed and adjusted for the sample weights and cluster that is used in the DHS data. Also, please be specific how the binary logistic regressions were adjusted for. Describe in detail how multicollinearity was accounted for.

**Response:** The data were weighted using the sampling weight variable (v005). A weight variable was generated by dividing the variable (v005) by 1,000,000. This was applied throughout the various stages of the analysis. The binary logistic regression was adjusted by using the enter mode in the mode where all variables were put in the model and run at the same time. In addition, we checked for multicollinearity and highly correlated variables such as mothers' place of residence and religion were excluded from the analysis because they were collineated.

22. Line 171 This contradicts your statement that 613 children were exclusively breastfed

**Response:** The entire results section of the revised manuscript has been modified (page 8, lines 158 - page 14, line 210).

23. Table 3 is very confusing. Review other PloS One articles for information on how to construct these types of tables

**Response:** We thank the reviewer for the observation. We have reviewed other PloS One articles and revised all tables in the revised manuscript.

24. Line 262-263 Your paper is specifically about exclusively breastfeeding and not maternal health; consider revising this statement to more accurately reflect your study

**Response:** We have modified the sentence to accurately reflect exclusive breastfeeding. We changed the phrase “maternal health” to “EBF” in the revised manuscript (page 14, line 215).

25. Line 271 Need reference after WHO

**Response:** We thank the reviewer for the observation. The statement has been referenced in the revised manuscript (page 15, line 223).

26. Line 275 Too vague. Consider giving specific examples on measures and policies that need to be strengthened. What types of interventions have worked well in other countries? There are examples of programs that involve partners to improve breastfeeding

**Response:** We have included specific programmes in Italy, Brazil and Taiwan, and their impact on both breastfeeding and exclusive breastfeeding in the revised manuscript (page 15, line 231-237).

27. Line 287-298 This needs to be explained more. Many studies find that higher maternal education is associated with formula feeding and non-exclusive breastfeeding. It is possible this is a statistical error based on sample sizes within the education variable or may be an error related to unaccounted for multicollinearity.

**Response:** We have revised the manuscript to account for multicollinearity. Variables such as maternal place of residence and religion were collineated and consequently, they were excluded from the analysis.

28. The discussion is mostly about mother's characteristics. The title and justification of the study was built on the premise of partner's characteristics. Consider revamping the study if you want to look at maternal characteristics as well. If the paper is about partner characteristics then the discussion needs to adequately reflect that. Discussion should also include specific recommendations to policy makers, public health professionals, and detailed recommendations on intervention design. What should be changed in Ghana because of the study results to improve exclusive breastfeeding in Ghana? Give examples of what has worked in other countries involving fathers and partners to improve breastfeeding. Could those be used in Ghana? What would be possible barriers? What type of future research should be done because of your study?

**Response:** We have included more other partner characteristics such as husband desire for children, religion, ethnicity, place of residence, partner attitude towards wife beating, and number of children by partner in the revised manuscript (page 6, lines 118-120). In addition, the discussion now reflects partner characteristics and recommendations on policy to health workers. Specific programmes involving men and women that have worked in other countries have been included in the discussion. In addition, specific programmes that could be also be adopted in Ghana have been added to the revised manuscripts. Also, future research has been indicated in the manuscript. See from page 14, line 213 to page 19, line 323.

29. Double check references to make sure they are accurate

**Response:** We have double check references to make sure they are accurate.

**Reviewer 2**

1. The objective of the article is to establish and comment on the pattern between exclusive breastfeeding and partner characteristics, however, the abstract does not mention this relationship as much as it needs to. There seems to be a greater emphasis on maternal characteristics instead which defeats the purpose. The authors need to bring out the effect of paternal characteristics more

**Response:** The abstract has been revised to include the influence of partner characteristics on exclusive breastfeeding (page 2, lines 18-36).

2. The list of paternal characteristics that influence breastfeeding is certainly not exhaustive, however, some key paternal characteristics have been left out of consideration. This includes – paternal religion, paternity leave, paternal ethnicity, children fathers have had, father’s place of residence, etc. These indicators are generally available in the DHS surveys and should be available in the data dump the authors obtained. Additionally, similar factors have been considered for mothers but not for fathers who are the primary subject of this study. I believe the author’s need to revisit their overall study objective and align the methodology of the paper accordingly.

**Response**: We thank the reviewer for the observation: We have included other partner characteristics such as husband desire for children, religion, ethnicity, place of residence, partner attitude towards wife beating, and number of children by partner in the revised manuscript (page 6, lines 118-120).

3. The entire discussion section beyond paragraphs 1 and 3 (page 22) talks only about maternal characteristics. The authors need to revisit this entire section.

**Response:** The entire discussion section has been modified to reflect the influence of partner characteristics and exclusive breastfeeding in the revised manuscript. See from page 14, line 213 to page 19, line 311.

4. Page 22, para 3, line 1 – The line states that “the findings indicate that partner’s occupation was the only characteristic of a partner that influenced the practice of exclusive breastfeeding”. This statement although true in the given context of the study is appreciated, would only be valid if supported with a more extensive assessment of other characteristics (mentioned in point 2).

**Response:** We agree with the reviewer’s comment. As a way of making the results robust, we have added other partner characteristics such as husband desire for children, religion, ethnicity, place of residence, partner attitude towards wife beating, and number of children by partner in the revised manuscript (page 6, lines 118-120).

5. The discussion section of a paper is meant to fit the results and observations of a study in the wider context of existing literature. Some interesting parallels can be made by drawing on other aspects of maternal and child health. For instance, are similar trends seen in the case of infant malnutrition, maternal health-seeking behavior etc.

**Response:** We thank the reviewer for the comment. We have highlighted in the revised manuscript that although Ghana’s EBF rate has declined, there has been improvement in other child health indicators such as stunting, wasting, and underweight (page 15, lines 226-229).

6. The authors can also add a paragraph in the introduction to explain the cultural context of breastfeeding in Ghana. Since breastfeeding often has social and cultural significance, it would help the readers understand if that might have any potential impact on overall breastfeeding practice in Ghana.

**Response:** We have added a paragraph in the introduction section which talks about the influence of interpersonal and community factors on exclusive breastfeeding in Ghana in the revised manuscript (page 4, lines 71-78).
