# Supplementary material for: The association between paternal characteristics and exclusive breastfeeding in Ghana
Source: PLoS One. 2021 Jun 3;16(6):e0252517. doi: 10.1371/journal.pone.0252517 (PMC8174696; doi:10.1371/journal.pone.0252517)
Supplement: Attachment — Submitted filename: Response to Reviewers.docx [file pone.0252517.s004.docx]

**Responses to the academic editor and reviewers’ comments**

**Academic Editor**

1. Please review your reference list to ensure that it is complete and correct. If you have cited papers that have been retracted, please include the rationale for doing so in the manuscript text, or remove these references and replace them with relevant current references. Any changes to the reference list should be mentioned in the rebuttal letter that accompanies your revised manuscript. If you need to cite a retracted article, indicate the article’s retracted status in the References list and also include a citation and full reference for the retraction notice.

**Response:** We have gone through all the references to ensure that they are complete and correct. We have removed two references (Asare et al. [38]; Sika-Bright and Oduro [46]) from the revised manuscript since they talked about the effect of mothers' education on exclusive breastfeeding. We have included new references (Ludvigsson and Ludvigsson [34]; Chudasama, Patel, and Kavishwar, [46]) in the revised manuscript to corroborate our finding that partners with low education are less likely to practice exclusive breastfeeding compared to those with no education. Also, we have modified the sentence “Therefore, formal education by itself may not necessarily empower a mother to practice EBF.” to read “Therefore, mothers whose partners have acquired some level of formal education may not necessarily be empowered to practice EBF.” Please see Line no: 252-253.

In addition, we have removed the reference Bhatta [48] from our revised manuscript since it did not support our argument in the manuscript. Also, we have deleted the reference Adsera [50] from the revised manuscript since we have deleted the argument it supports from the revised manuscript.

38. Asare BY-A, Preko JV, Baafi D, Dwumfour-Asare B. Breastfeeding practices and determinants of exclusive breastfeeding in a crosssectional study at a child welfare clinic in Tema Manhean, Ghana. International Breastfeeding Journal (2018) 13:12 2018;13(1):12.

46. Sika-Bright S, Oduro GY. Exclusive breastfeeding practices of mothers in Duakor, a traditional migrant community in Cape Coast, Ghana. Journal of Global Initiatives: Policy, Pedagogy, Perspective. 2013;8(1):87-102.

34. Ludvigsson JF, Ludvigsson J. Socio‐economic determinants, maternal smoking and coffee consumption, and exclusive breastfeeding in 10 205 children. Acta Paediatrica. 2005;94(9):1310-9.

46. Chudasama RK, Patel PC, Kavishwar AB. Determinants of exclusive breastfeeding in south Gujarat region of India. Journal of Clinical Medicine Research. 2009;1(2):102.

48. Bhatta DN. Involvement of males in antenatal care, birth preparedness, exclusive breast feeding and immunizations for children in Kathmandu, Nepal. BMC Pregnancy and Childbirth. 2013;13(1):1-7.

50. Adsera A. Differences in desired and actual fertility: An economic analysis of the Spanish case. Bonn: IZA; 2005.

**Reviewer 2**

1. Line no. 22 – Correction to “This cross-sectional study used data from the 2014 Ghana Demographic and Health Survey”.

**Response:** We thank the reviewer for the comment and observation. This has been corrected in the revised manuscript. Please see Line no. 22.

2. Line no. 30 – Replace “desire” with “desired”

**Response:** The word “desire” has been changed to “desired” in the revised manuscript. Please see Line no. 30.

3. Line 78-79 – Partner is defined according to empirical evidence. However, it must also be mentioned if it aligns with the definition as per the DHS data

**Response**: We thank the reviewer for the comment. We have revised the definition of a partner in the revised manuscript to align with the DHS definition. A partner is defined as “a man who is either currently married or living with a woman”. Please see Line no. 79.

4. Was the sample representative of the ethnic, religious, occupational standards for Ghana? In either case, this should be mentioned in the methodology and then inference accordingly made in the discussion.

**Response:** Ethnicity, religion, and occupation are aligned with the standards for Ghana. We have included the statement “All variables and their categories used in this study align with the standards for Ghana.” in the revised manuscript to that effect. Please see Line no. 145-146.

5. Line 231- 232 – Why is neonatal mortality being mentioned here? It hasn’t been talked about any where in the passage. How is this adding to the overall discussion?

**Response:** We thank the reviewer for the comment. Neonatal mortality is very important with regard to exclusive breastfeeding. In that non-exclusive breastfeeding has a consequence on mortality. We have therefore modified the sentence “It reduces infant mortality and morbidity, enables infants to crawl early, enhances growth and cognitive development, and reduces the risk of childhood obesity [3-7].” to read “It reduces neonatal and infant mortality and morbidity, enables infants to crawl early, enhances growth and cognitive development, and reduces the risk of childhood obesity [3-7].” in the introduction section to that effect. Please see Line no. 46-47.

6. Line 241- 246 – The authors need to establish who the primary focus of the study is – partners or mothers? If the objective is to compare the two, that needs to be brought out better

**Response:** We thank the reviewer for the observation and comment. Partners are the primary focus of this study. We used mothers’ education to explain partners’ education. We have therefore modified it in the revised manuscript. Please see Line no. 247-249. In addition, we have modified the sentence “It was also evident in the findings that the likelihood to exclusively breastfeed was lower among Muslim mothers compared to Christian mothers.” to read “It was also evident in the findings that the likelihood to exclusively breastfeed was lower among mothers whose partners were Muslims compared to those whose partners were Christians.” Please see Line no. 259-260.

7. Line 251 – 252 – Is there any evidence to support this statement?

**Response:** The statement “Therefore, partners who desire more children may discourage the practice of EBF to ensure their fertility desire is accomplished.” is speculative and we have deleted it from the revised manuscript.

8. Line 255 – 262 - Is there any evidence to support these statements? Was the survey done during Ramadan? The relevancy of these statement is not clear or justified.

**Response:** We thank the reviewer for the comment. We have modified the paragraph in question as follows: “It was also evident in the findings that the likelihood to exclusively breastfeed was lower among mothers whose partners were Muslims compared to those whose partners were Christians. This finding is consistent with previous studies [23]. The Holy Quran encourages breastfeeding among Muslims [48]. However, the reasons why mothers whose partners were Muslims are less likely to exclusively breastfeed compared to those whose partners are Christians could not be explained in the present study and therefore further studies using qualitative approaches could explore the probable reasons.” Please see Lines no. 259-265.

9. Line 266 – Which finding do the authors mean when they say “this finding”

**Response:** We thank the reviewer for the comment. We have revised the manuscript and deleted the section “This finding however contradicts our finding that mothers whose partners desire more children were less likely to practice EBF compared to those whose partners desire fewer children. A probable reason for the discrepancy in the findings is that there is a difference between actual and desired fertility. Desired fertility may not necessarily translate into actual fertility due to several factors including labour market and economic conditions [49].”

10. Line 271 – 287 – How does this fit into the objective of the study – understanding partner characteristics that impact EBF?

**Response:** We thank the reviewer for the comment. Although our study focused on the influence of partner characteristics on exclusive breastfeeding, mothers and infant characteristics also influence exclusive breastfeeding. Consequently, we controlled for these characteristics. Since the mother’s antenatal care visits and age of infant were found to be significantly associated with exclusive breastfeeding in this study, we, therefore, discussed it in the “Discussion Section”.

11. Of the ~40 AOR calculated, majority are insignificant. The authors should include a segment discussing these observations.

**Response:** We thank the reviewer for the suggestion. We have discussed the non-significant paternal characteristics in the “Discussion Section”. Please see Line no. 269-278.
